# Making the Matrix

**Published:** 1904-07

**Authors:** Lee K. Stewart

**Affiliations:** Chicago.


### MAKING THE MATRIX.

By Lee K. Stewart, D. D. S., Chicago.

(Written for *The American Journal of Dental Science.*)

With the cavity prepared and easy access obtained, if necessary by some method of separation, or the forcing of the gum away with gutta-percha temporary stopping or cotton, the next step is the making of the matrix.

The artistic in porcelain filling is founded upon good cavity preparation and matrix adaptation. Inlays are not quickly made, and to get beautiful and lasting results, patience and skill must be exercised in fitting the metal foundation. Too often this part of the operation is slighted with the expectation that the cement will fill the spaces.

This is a very great mistake, for it both makes a much weaker filling and the mass of cement alters the color of the porcelain.

With experience the operator acquires skill sufficient to fit the matrix, in the simpler cases, directly to the cavity.

It is not the purpose of this paper to treat of these cavities but the method advised will be applicable in all cases, whether gold or platinum is used, and is as follows:

Thoroughly rub into the cavity cotton covered with soap-stone or talcum powder, and then carefully wipe out all of this material.

Take an impression of the cavity with one of the quick setting cements mixed quite thickly and firmly forced into place. When hard, remove and you have a good cement impression of the cavity. Surround the impression with wax and fill it with Fellowship amalgam, being careful to pack it in small quantities and with considerable pressure. Allow the amalgam to harden and separate. If the cement impression is of such shape that there is danger of breaking the edge of the amalgam model, immerse the mass in aqua ammonia for several hours before separating.

Mount that amalgam model of the cavity with modelling compound, in the cup of the Brewster water bag swedging press. Burnish the metal for the matrix loosely into the floor of the cavity, cover the matrix with some moistened absorbent cotton and put under slight pressure in the press. Remove the cup and cotton, burnish out any folds in the metal, anneal and repeat, using more pressure each time until the matrix fits the model. After the metal has been thoroughly swedged into place, carefully anneal and again put under pressure as before. This last is very important in order to prevent the drawing of the metal in the fusing of the porcelain and gives an annealed matrix which perfectly fits the amalgam model of the cavity.

If gold is used and consequently a low fusing porcelain, fill the matrix to the desired contour and fuse cool and place the inlay in position on the model. The shrinkage in firing will be considerable and the outer edge of the matrix will be free from the porcelain, so you can burnish the margins again in the model if necessary. Add porcelain leaving the outer edge of the matrix exposed and fuse once more. At the next sitting of the patient, place the inlay in position and burnish carefully the edge of the matrix which you have left uncovered to the outer walls of the cavity, add the necessary porcelain and complete. A strip of rubber dam drawn over the inlay and tooth holds it in position, and facilitates the final fitting.

In using platinum and a high fusing porcelain with a higher fusing foundation body, after the matrix has been completed fuse the proper amount of foundation body into the matrix leaving the margins exposed. If the case is a large one it may be advantageous to make two bakings of the foundation body. After this is completed, fit the partially made inlay directly to the cavity in the tooth, as with the gold matrix and finish.

There are many advantages in making the matrix in this way, as you get the benefit of the amalgam model and later complete the matrix in the tooth cavity. It is first fitted to as good a model of the cavity as can be made and then put into the cavity in the tooth, after enough porcelain has been fused in the matrix to firmly establish the shape of the inner part, and the outer walls are easily and accurately burnished to the walls of the tooth. This gives an opportunity to make any changes that may be found necessary and to test and perfect the fit of the matrix. In describing this method it has been essential to say something of the first fusings of the porcelain.

---

### SELECTING THE SHADE.

By Geo. W. Schwartz, M. D., D. D. S., Chicago.

(Written for *The American Journal of Dental Science.*)

